# 

**Published:** 1842-12

**Authors:** 


					EDWARD MAYNARD, M. D.
H. M. HAYDEN, M. D,
M
ELISHA TOWNSEND, :
VEBNON C0Y&OR, M. B. *
i ISAAC t GREENWOOD, M. 0.
G. G. BREWSTER, , : . ""
|'' $ ? ?
IjSin^dj. ip. '-2
LUDOLPH PARMLY.M. D.
EDWARD TAYLOR, M. D.
C. 6. BREWSTER, M* D. wmm-
LEONARD KOECKER, M. D.
LEWIS KOPEK, M. D. PhU?de.nhi,
jfc A BURR, M. D. Philadelphia.
E. BAKER, M. D.
HORATIO N. FENN, M. D.
ALEXANDER NELSON, M. D.
B. A. EODRIGUES, M. D.
mm
HDP aOGEfiS,
ENOCH MOYES, M.
Vi?&$fc- jet.
SUBSCRIPTIONS TO THE SECOND VOLtiME.-Our readers
rence to the Prospectus on the fourth page of the cover, that the terms of s
in advance The Journal having become the property of the Ameri tfaat this
5 ? *******
have signified their wish to Continue their subscription, without having paid the amount, may
attach any blame to the editors for not furnishing them with it, until they shall have done so,
OFFICERS AI|P MEMBERS OF THE AMERICAN SOCIETY OF
SURGEONS.?A catalogue of the Officers and Members of this Society has just
lished by the Recording Secretary, by order of the same. It is beautifully executed,
in a convenient form for framing. Every member should have On
cured by application to the above named officer, on parchment, silk,
by transmitting tojum one dollar, the price fixed by the Society.
DIPLOMAS.?The American Society of Dental Surgeons is
tifully executed diploma plate, and will furnish such of its
parchment diploma. Any member, therefore, who may wish to j
nished with the san&j'W transmittu
Receipts of Subscriptions to the Second Volume of the American Journal
and Library of Dental Science.
Harvey Sellers,  $5 00
Dr. Lewis Roper,  5 00
Dr. Robert Yandervoort,.    ... 5 00
:? ; 3 vv
7
M.
m
IBifl
v; *    S 00
S).    #... 5 y
V -,.n? ., ' K All
5 00
Dr. E. B. Gardette,    6 00
9 UO
Robert Erity  ? ? i ? ? * *> *
Dr. Robert Vandervoort,.      5 uu
Alcock, James....... ,    . iv 5
Ashley, R. V....   5 00
Brown,B. B..,   .**
Ballard, Uto. W.??????? ?i ????? ? ? ?????????????? &
Blandioff, S. ,M.???????????????????????????? 5 0&
Claggett, H.           5
Clute, Nicholas ? ? ? t ? ?        ? ? ? 5
I
5 00
Bode?* Neherailah.- ? ? ????? ???????? #>> ?.# ??? ^ ? W
Dinsmore, J. P. ...?.............,. 5 00
Goddard, Win* HL t ? ? 5 00
Gunning, S. B,   5 00
3? *? I
C"? ?;V ? V.. * -
??ale, Jiid     o uu
Jewett. U. H. >  ..  ????? ? *? ??? ? ? ?
.Charles............ ...    5 00
N...  .....A... ?5 00
litb, Swifts????????. i >?? ? ?        ???? o 00
tl - #1 > T1 * *>.-'??. f v" - ->V- / e nn
?eetlana, Jt56nj? o uu
IB* w* W.?? ? ? ? ?,? ? ? ??*???????? # # # ? ? ? v uU
fw-' \ -r. -ft aa.
? ? ,? ? ? ?.??'*? rt ? ?????? ? ? ????????? ? i ? w ""
v..    9 uu
6 00
Thornton, J. T\..? i ???????????? t ?? t ? i ?? i ? 5 00
3X0> W I* ??????? ?????????#????????????? ??? 8 UO
r, E. I)* ? ?????? t ?????????????????*?? ? ??5 00
'w&
?jsuing a list of those who hate pg
[ answer in place of sending a receipt to each person.
APPLICATIONS FOR MEMBERSHIP;
last session of the American Society of Dental Surgeo:
inted in the cities of Boston, New York, Philadelphia, and ;
>mmitfee8,
number.
riffl TOi ifWiV? -?-J3,v*3?Bk-aK jarSn&VBtmak ?>> ? ?<?.
EDITORS' PROSPECTUS
M mmwrn..
o:
this Journal bas become the organ of the American, Society of Dental Surg
has been somewhatwodifiedT it may be consider^ as haviri? entered upon
:s existence) and its former publishers congratulate themselves and the prolesr
>rtune. Its perpetuity is now considered as settied. The profession will always
?evoted to its Interests, and who is better able to conduet it, than the only regular
extensile association of dental sargeons in the world- We repeat* that we cot
and our protessional Dretnreti on an event so auspicious to tne science ot dental
formation of a national society and the transfer of the journal to that body. The
regularly issued, whether its circulation be?co-extensive with the profession or not.
tion should &e limited, the issues can be made to correspond with the demand, so thj
society may be kept wholly within its means. M - -?
American Journal and Library of Dental Science, is now proposed to
fZ'Ji
ground, and will hereafter solicit no favours from those members of the prole
to the dissemination of periodical information. To others who are favourably
nity of knowledge, this journal will not fail to commend itself, as one of the n
of exposing the impositions of quackery, and imparting to honorable pretensions
mation whick their usefulness and talents should seeure.
pr.ce of subscription moy be poid !o tho tiossunTof'tfie1^' flfS *d"
City, or to either of the editors or anv of the ,'' y' r Leonard Mackall,
the Journal. Advance pay^enT s reqied 7^
of the first volume in unexpected embarrassment, if not in animate loss.
To save postage, the agents in all the large cities of the United States, and Great
lrnished with copies to supply subscribers, by such conveyance as they shall ]
where it can be done, even individuals may receive their numbers by private com
With these few explanatory remarks, we commit our work to its coming destiny,
the profession that no efforts wilt be spared to render it every way worthy of the
which its pages wift be exclusively devoted.
?
CHAPIN A. HARRIS,
SOLYMAN BROWN,
LEONARD MACKALL.
a*-V v . 1
Besides the ColIalMrators, the following gentlemen are regularly authorized
OP THIS JOUBHAL.
JURY & Souew, Boston.
M'CoifKELL, M. D.... .Richmoud, Va.
Atbp, M. 0.......... .Columbus, Ga.
|^?js<6Tow Paxmty, New Orleans.
>, M. D ...... .Macon, G?.
& AvEftY,..; Columbia, s. u.
lb GiDNur,.... ... . Manchester, Ear.
K % Savannah, Geo. J
Ferguson, Louisville, Ky.
James Taylo*, M. D.... . . .Cine
Nicholas Glut*,..;...... ,.l.ouis\
J. N- F-R.ni K, .??.*?????? ??. ? Pt
E. $* Dunning,. .  ;
Ben.t. 8tbickland,
B.B Baowu, M.D..
W. E. Scott, D. D. S   .1
S. M. Shepherd
W. W. H. Thackston, D.D. S. Farmt
EdwaUd Taylor, M. D. ..,. .Natchez,

				

